# The Less We Eat, the Longer We Live: Can Caloric Restriction Help Us Become Centenarians?

**DOI:** 10.3390/ijms23126546

**Published:** 2022-06-11

**Authors:** Tamara Dakic, Tanja Jevdjovic, Predrag Vujovic, Aleksandra Mladenovic

**Affiliations:** 1 Department for Comparative Physiology and Ecophysiology, Institute for Physiology and Biochemistry “Ivan Djaja”, Faculty of Biology, University of Belgrade, Studentski trg 16, 11000 Belgrade, Serbia; tamara.dakic@bio.bg.ac.rs (T.D.); tanja.jevdjovic@bio.bg.ac.rs (T.J.); predragv@bio.bg.ac.rs (P.V.); 2 Department of Neurobiology, Institute for Biological Research “Sinisa Stankovic”—National Institute of Republic of Serbia, University of Belgrade, Bul.D. Stefana 142, 11000 Belgrade, Serbia

**Keywords:** caloric restriction, centenarians, longevity, health span, metabolism, insulin sensitivity, signaling pathways

## Abstract

Striving for longevity is neither a recent human desire nor a novel scientific field. The first article on this topic was published in 1838, when the average human life expectancy was approximately 40 years. Although nowadays people on average live almost as twice as long, we still (and perhaps more than ever) look for new ways to extend our lifespan. During this seemingly endless journey of discovering efficient methods to prolong life, humans were enthusiastic regarding several approaches, one of which is caloric restriction (CR). Where does CR, initially considered universally beneficial for extending both lifespan and health span, stand today? Does a lifelong decrease in food consumption represent one of the secrets of centenarians’ long and healthy life? Do we still believe that if we eat less, we will live longer? This review aims to summarize the current literature on CR as a potential life-prolonging intervention in humans and discusses metabolic pathways that underlie this effect.

## 1. Introduction

Scientific studies for increasing human lifespan have been present since the early XIX century [1]. Caloric restriction (CR), restricted nutrition without malnutrition, was undoubtedly proven to extend lifespan in short-lived species (reviewed in [2]), but it is still debatable whether it has the same effect on human lifespan. One approach to assessing the effect of CR on human lifespan is to analyze life expectancy in regions where certain forms of CR are practiced as a daily habit. A specific, religiously-oriented food restriction that can be considered a form of CR was well known for hundreds of years (reviewed in [3]). Many religions incorporate fasting periods; the best known are Islamic Ramadan, the three principal fasting periods of Greek Orthodox Christianity (Nativity, Lent, and the Assumption), and the Biblical-based Daniel Fast. There are several studies examining the effects of Islamic Ramadan on health, as well as Greek Orthodox Christian fasting. Although several beneficial metabolic changes were recorded under such a regimen (reviewed in [4]), at this point it is impossible to compare the results of the studies and to draw any conclusion regarding the effects of religious fasting not only on lifespan but also on health span. Many variables that exist between different cultures and religions, such as the schedule of fasting, the lack of medical records, food choices and eating habits, and the lack of reliable scientific data, exclude the possibility of establishing a correlation between religion-based food restriction and average lifespan.

### 1.1. Populations of Centenarians

The alternative approach is to study the longest living populations, namely centenarians, and to analyze their dietary habits. Centenarians are people who are 100 or older, while those who live to be 110 years or more are considered to be supercentenarians. It is estimated that this is a growing population [5]. The number of centenarians increased from approximately 417,000 in 2015 to 573,000 in 2020. Moreover, it is predicted that their population will reach a number of 19 million in the year 2100 (source: https://www.statista.com/statistics/996597/number-centenarians-worldwide/, accessed on 22 May 2022). Namely, it seems that most babies born in developed countries since 2000 will become centenarians (reviewed in [6]).

Most centenarians reach such an old age because they manage to postpone the major age-related and life-threatening pathologies (e.g., ischemic heart disease, stroke, chronic obstructive pulmonary disease, cancer, respiratory infection, type 2 diabetes, osteoporosis, and dementia). Protein signature data from the New England Centenarian Study suggest that centenarians age slower than other humans [7]. The fact that centenarians also suffer from severe age-related diseases and/or disabilities, but do not die from them at the age that the majority of humans do, points to an improved regulation of resistance and an increased intrinsic capacity to respond to minor stresses of daily life (i.e., resilience) [8,9]. Centenarians do not escape normal aging markers, but rather acquire them much later in life than the average population, keeping the rate of age-related physiological decline, or of the development of age-related diseases or syndromes, slow enough to be counterbalanced by their resilience. This fact sheds light on the importance both of genes that control body resistance (reviewed in [10]) and of environmental factors such as caloric restriction, known to be able to mediate resilience [11].

In addition to a genetic predisposition to becoming a centenarian (reviewed in [12,13,14,15,16]), lifestyle in general and nutrition in particular play a significant role in achieving this milestone. A careful observation of the major centenarian populations worldwide points to a specific nutritional strategy they use.

Worldwide demography of centenarians shows that their number is negligible in low-income countries, while the shares of male and female centenarians living in high-income European countries is decreasing. At the same time, middle-income countries for the first time are experiencing an increase in the oldest population (reviewed in [17]). However, it is considered that very few countries can provide long-term reliable data regarding centenarians and those are mostly the Nordic and Western European countries and Japan. Therefore, the best approach is to focus on so-called Longevity Blue Zones (LBZ), well-studied specific geographical regions characterized by the highest number of centenarians [18,19]. Blue zones are relatively limited and homogenous geographical areas with exceptionally high longevity in their populations. The most famous LBZs are Okinawa (Japan), Nicoya Peninsula (Costa Rica), the island of Ikaria (Greece), Sardinia (Italy), and Loma Linda in California [19]. Among nine evidence-based lifestyle principles (strong family connections and close social engagements, meaningful life purpose, low-level physical activity throughout the day, staying stress-free, moderate alcohol consumption, belief in a higher power) that people in these areas apply to live longer and more healthily, the most important one appears to be nutrition. A more detailed analysis of centenarians’ nutritional habits revealed that centenarians usually consume high amounts of legumes, avoid overeating, and apply caloric restriction and/or fasting. Three out of five blue zone populations were more extensively studied regarding their caloric intake: Okinawa, Sardinia, and Ikaria. The most relevant publications on centenarians discussed in this review are listed in Table 1.

#### 1.1.1. The Okinawan Population

Okinawa’s population of centenarians is perhaps the most studied one. Although there were some doubts regarding the actual number of centenarians in Japan, subsequent validation confirmed the high centenarian prevalence in Okinawa [38]. Further studies revealed a genetic aspect of longevity, as siblings of Okinawan centenarians have an over three times greater likelihood of becoming centenarians [20,39]. This population is genetically distinct and shares several characteristics of a population isolate; as a consequence of genetic drift, natural selection, and population bottlenecks that are present in such populations, they are considered prone to developing extreme phenotypes such as longevity.

However, an investigation of Okinawans’ nutritional habits uncovered a strong impact of nutrition and caloric intake as well. Available epidemiological evidence indicates that CR may have already contributed to an extension of average and maximum lifespan in Okinawans who appear to have undergone a mild form of prolonged CR for approximately half of their adult lives [35]. One of the first studies conducted on Japanese centenarians showed a negative correlation between total energy intake and the proportion of centenarians [30], indirectly indicating that low caloric intake is a requirement for achieving longevity. The studies also reported relatively low caloric intake in Okinawan school children (62% of the calories of other Japanese school children in the early 1960s) [24] and the Okinawan adult population (83% of the Japanese average) [25] and hypothesized that this could account for their healthy longevity and significantly lower risk for age-associated diseases than found in other Japanese. A more recent and detailed investigation of archived population data on the elderly (aged 65-plus) cohort of Okinawans confirmed that the Okinawan population has a low caloric intake and negative energy balance at younger ages, little age-associated weight gain, lifelong low body mass index (BMI), relatively high plasma dehydroepiandrosterone (DHEA, a marker of longevity) levels at an older age, low risk for mortality from age-related diseases, and survival patterns consistent with extended mean and maximum lifespans [36]. A subsequent study that made a comparison between Okinawans and Americans showed that Okinawans had much lower caloric intake than Americans and could be considered mildly calorically restricted (10–15%) at younger ages relative to their estimated energy requirements [37]. In addition, dietary and phenotypic data consistent with CR in Okinawan septuagenarians (population between 70 and 79 years old) and centenarians were reported [22].

#### 1.1.2. The Sardinian Population

Italy is a world-leading country regarding research on centenarians; multidisciplinary and multipurpose research projects AKEA 1 and AKEA 2 applied several different approaches to study the genetic and environmental basis of Italian longevity [34]. These studies provided valuable data regarding human longevity and healthy aging. Similarly to Okinawans, it seems that some genetic factors are associated with human longevity in Italy (reviewed in: [21,28,32]). Importantly, the probability to become a centenarian occurs in a gender-dependent manner. Namely, an exceptionally high prevalence of male centenarians was detected in this Blue Zone; the high male/female ratio for validated centenarians seems to be unique to this island and points to the possibility that environmental characteristics and/or genetic factors exert their favorable effect more strongly in men than in women [34]. A recent study assessing global cognitive efficiency, life satisfaction, lifestyle, food intake, and perceived physical health in Sardinian centenarians indicated that healthy nutritional habits and a physically active lifestyle are associated with this exceptional longevity [23]. A more detailed analysis of dietary patterns in this Blue Zone revealed that specific nutritional intake plays a role, with vegetable consumption showing a low correlation with health indicators, while meat (poultry) intake was associated with better physical performances [33]. High consumption of cereals was also reported. Although this study analyzed the average intake of 15 types of foods, it did not investigate caloric intake in the participants. Likewise, a study of the oldest people in Ogliastra (Sardinia) and from the Nicoya peninsula only revealed that centenarians from these regions consume a mainly plant-based diet [29] without giving precise insight into their nutritional caloric score. Therefore, we can only indirectly conclude that longevity in these areas could be related to a low(er) caloric intake, which usually complements healthy dietary patterns.

#### 1.1.3. The Ikarian Population

Ikaria Island in Greece belongs to the group of places with the highest life expectancies in the world. The inhabitants of Ikaria Island represent an isolated rural group and have one of the highest longevity rates. Several studies were conducted to analyze and reveal the reasons lying behind this high proportion of centenarians. Sociodemographic and lifestyle statistics were gathered, as well as biological analyses. Again, daily physical activities and healthy eating habits were reported within the oldest population [31]. Fascinatingly, in contrast to the previously discussed populations of centenarians from Japan, the study related to the Ikarian population showed that only 42.2% of participants were normal weight, while 40.6% were overweight and 17.2% were even obese [26]. Unfortunately, this study did not include dietary and nutritional assessments; therefore, we miss more detailed insight into the nutritional habits of this population. Similarly, the overall assessment of dietary habits of Ikarian centenarians, evaluated through a special diet score, did not provide data concerning average caloric intake, but only indicated greater adherence to the Mediterranean dietary pattern, and consequently, healthier dietary habits [31]. However, the results of a subsequent study [27] showed that existing data regarding the Mediterranean diet as a determining factor leading to longevity are less convincing in comparison to other environmental factors, such as family solidarity, social interaction, and physical activity.

### 1.2. Human Studies

Apart from the epidemiological data presented above, several other human studies noted favorable changes induced by CR, particularly those related to cardiovascular and glucoregulatory functions. Although these changes do not provide a definitive answer to the dilemma of whether CR prolongs lifespan, they still imply that CR has a beneficial impact on longevity (reviewed in [40,41]).

A relatively short-term study (10 weeks) conducted by the Toxicology and Nutrition Institute in the Netherlands (TNO) included 16 male participants exposed to a 20% CR that were compared to 8 *ad libitum* control subjects (all 35 to 50 years old) [42]. CR-exposed subjects lost weight (mostly fat mass) and lowered their blood pressure, but showed a trend toward elevated high-density lipoprotein (HDL) levels that, interestingly, correlated with weight loss (reviewed in [43]). A significantly longer study was Biosphere 2 [44], which represented a voluntary experiment in a closed ecosystem in the Arizona desert and encompassed eight participants (four males and four females, 30 to 40-year age range) exposed to a 2-year 30% CR. The study provided several interesting results that further support the health benefits of a low caloric intake, but did not deliver any data regarding CR impact on the lifespan of the participants. Apart from the expected marked reduction in body weight, fasting glucose, blood pressure, serum cholesterol, triglyceride levels, and insulin levels, total plasma HDL levels were decreased in the Biospherians [43,45,46].

#### CALERIE^TM^ Study

The most extensive scientific assessment of the effects of reducing energy intake conducted on humans so far was the Comprehensive Assessment of Long-term Effects of Reducing Intake of Energy (so-called CALERIE^TM^ study)—a multicenter study designed and established in an attempt to determine the biological effects of caloric restriction in healthy, normal-weight, and slightly overweight participants [47]. There were no previous clinical studies of non-obese individuals who attained CR; thus, this study represents an enormous improvement in understanding what the effects of CR could be in young, non-obese healthy subjects, and it generated an extensive collection of data and samples.

CALERIE Phase 1 (CALERIE 1) tested the effects of 6- and 12-month-long CR (10% to 30% energy-deficit diets) among healthy, middle-aged adults who were overweight but not obese. Energy deficits were achieved through reduced food intake, increased exercise energy expenditure, or a combination of the two. The results indicated that fasting insulin and body temperature, two longevity biomarkers, were reduced by 6-month CR in humans [48]. A prolonged study (12-month CR) showed that CR effectively reduced body weight and adiposity [49], and improved glucose tolerance and insulin sensitivity [50], but was not more efficient in promoting the aforementioned effects than physical exercise.

CALERIE Phase 2 (healthy young to middle-aged men and women with a normal BMI exposed to 25% CR) provided valuable insight into time-dependent changes in adherence to this dietary regiment, body weight loss, and appetite modulation over two years. The study revealed that, although this mild CR negligibly affected appetite, adherence to such a regimen declined after 20 weeks, while weight loss occurred until approximately week 60 and then plateaued [51]. The study further showed that multiple cardiometabolic risk factors, such as plasma low-density lipoprotein (LDL)-cholesterol, total cholesterol to HDL-cholesterol ratio, and systolic and diastolic blood pressure were all reduced. Moreover, insulin sensitivity index and metabolic syndrome score also improved considerably [52]. In addition to that, cognitive performance was improved and resting metabolic rate was decreased in individuals exposed to CR [53]. Apart from the fact that CALERIE demonstrated the feasibility of sustained CR in humans (for at least two years), it also pointed to numerous beneficial effects on body mass composition, including a preferential loss of adipose tissue over loss of muscle and organ tissue [54].

The effects of CR on the lifespan of CALERIE participants are yet unknown, but there are indications that this regimen may favorably affect longevity as well. The improvement in two previously reported robust biomarkers of longevity (fasting insulin and core body temperature) was detected in non-obese participants after six months of CR [48]. Additionally, a metabolic change (a larger decrease in energy expenditure than expected based on metabolic mass loss) associated with lower thyroid hormone concentrations was documented in this study, further supporting the beneficial effects of prolonged CR on longevity. This is in alignment with previously recorded changes in body temperature and insulin level in CR monkeys, long-lived men [55], and self-selected practitioners of CR [56]. Interestingly, a reduction in DNA damage/fragmentation was detected, but there were no changes in protein carbonylation [48], previously found to be induced by CR in non-human primates [57] and obese humans [58], and known to be associated with longevity.

Lastly, it is worth mentioning that CALERIE participants did not experience adverse effects on mood, cognition, hunger, or sexual function, or serious adverse clinical events, apart from transient anemia and bone mineral deficits [59].

### 1.3. CR Impact on Longevity in Long-Living Primates

The available evidence in nonhuman primates suggests that although it is probable that CR increases the lifespan and/or health span, the degree of this increase is undetermined [60].

The first study of CR’s impact on longevity in long-living primates, in the Intramural Research Program of the National Institute on Aging (NIA), began in the late 1980s [61], while a subsequent study at the University of Wisconsin, Madison was initiated in the early 1990s [62]. Both studies were followed by a so-called “acute” or “invasive” study on monkeys subjected to shorter bouts of CR with the main goal of elucidating the biological mechanisms by which CR exerts its beneficial effects. Two additional studies, at the University of Maryland and at the Wake Forest University School of Medicine, were undertaken to examine the effects of CR on obesity, diabetes [63], and cardiovascular diseases [64].

Although certain aspects of experimental design differed between the two initial studies (NIA and Wisconsin), both investigated the effects of a 30% reduction in calorie intake on parameters of health, morbidity, and mortality. Data from these studies showed strong consistency with rodent studies regarding physiological responses to CR. Plasma insulin levels were reduced in CR monkeys and insulin sensitivity greatly improved. Furthermore, a reduced plasma glucose level was detected, which altogether indicated that CR could potentially prevent the development of adult-onset diabetes during aging. In addition to that, changes detected in other health parameters (decreases in % of body fat, plasma triglyceride and cholesterol levels, and mean arterial blood pressure) suggested that CR monkeys were less likely to develop cardiovascular disease, although those changes were more prominent in females, indicating that the effects of CR were gender-specific (reviewed in [43]).

Analyses of numerous potential biomarkers of aging in rhesus monkeys included in the NIA study offered additional evidence that CR might slow down aging. The markers included in this study were serum glutamic oxalacetic transaminase, alkaline phosphatase, total protein content, globulin levels, blood urea nitrogen to creatinine ratio, phosphates, and perhaps most importantly, plasma DHEAS. DHEAS was shown to protect against many age-associated diseases and its plasma levels declined 30% more slowly in CR animals in comparison to ad libitum-fed controls (reviewed in [43]).

However, the effects of CR on the lifespan were inconsistent. The Wisconsin study found that monkeys on a calorie-restricted diet had a significantly longer lifespan [65,66]. The NIA team, however, found no significant difference in survival, although a trend toward prolonged lifespan was noted [67]. This discrepancy most likely resulted from marked differences in dietary composition between the two centers, and perhaps more importantly, from differences in the subjects’ ages at the moment of CR commencement. The NIA study was conducted on monkeys at either young (before adulthood) or more advanced ages, while the Wisconsin study started at the adult stage. A very important conclusion arises from this observation, pointing out that the beneficial outcome of CR depends on the subject’s age at which CR is introduced. We detected something similar in experimental rodents. Namely, we found that late-onset CR worsened cognitive performance and increased frailty levels in female rats [68,69,70]. However, in contrast to the rodent studies, where decreased food consumption was shown to be beneficial from the early to adult stages and detrimental after late adulthood, it seems that beneficial effects in primates could be achieved only if CR starts after the subject reaches adulthood.

## 2. Physiological Processes Underlying Positive Effects of CR on Health and Lifespan

Although there is a consensus among the experts that aging can be slowed by dietary/nutraceutical interventions [71], considerable variations in CR-induced effects on the lifespan were documented (Table 2). Namely, CR affects lifespan in a species-specific manner, but differences in response were also noticed within species depending on CR extent and age of introduction [72]. This imposes the question of what accounts for this discrepancy, and what mechanisms are involved in CR-induced effects in short and long-lived species.

Thus, the other important question we wanted to address in this manuscript is what biological mechanism(s) underlie the life-prolonging effects of CR.

The findings from the GWAS/CLHLS (genome-wide association study/Chinese Longitudinal Healthy Longevity Study) support the hypothesis that, among other factors, metabolic changes driven by diet may play a pivotal role in longevity [73]. The link between metabolism, CR, and longevity is further supported by findings that CR led to the changes in the expression of genes that are classified both as candidate longevity genes and as key factors involved in metabolic processes (AP3K5, FLT1, PIK3R1, SIRT7, and SIRT5) [74].

Although these mechanisms are still not fully elucidated, some factors were recognized to contribute to lifespan extension mediated by CR. Namely, centenarians are characterized by a healthy phenotype that is largely similar to that found in adults following a CR diet, which includes optimal metabolic and endocrine parameters, reduced inflammation, and increased diversity in gut microbiota (reviewed in [75]).

### 2.1. CR, Insulin Sensitivity, and Longevity

Decreased insulin sensitivity is one of the characteristics of aging and thus represents a risk factor for numerous age-related disorders, such as type 2 diabetes, hypertension, and cardiovascular diseases [76]. In many studies that investigated CR and other interventions for lifespan extension, reductions in blood glucose and insulin levels were observed [77,78]. Therefore, it is assumed that these changes may help slow down aging by decreasing the rate of protein glycation [79]. In alignment with this is the observation that centenarians and semi-supercentenarians have reduced circulating insulin and fasting glucose levels [75].

Insulin resistance causes hyperglycemia and subsequently hyperinsulinemia. Since elevated plasma glucose is known to have deleterious consequences on health, experiments on male mice overexpressing glucose transporter 4 (GLUT4) tested the importance of decreasing glucose levels as an anti-aging action of CR [80]. Reducing circulating glucose via GLUT4 overexpression did not alter lifespan. However, a 40% restriction of food intake favorably affected longevity (approximately 25% extension) independently of genetic status [80]. This suggests that decreased blood glucose is not an important factor in mediating CR’s effects on the aging process.

On the other hand, it seems that decreased systemic insulin contributes to health span promotion and lifespan extension. Namely, a study on female mice with full or partial expression of *Ins2* (*Ins2*^+/+^ and *Ins2*^+/−^, respectively) showed that a lifelong moderate reduction in basal insulin levels improved insulin responsiveness in aged animals [81]. Moreover, *Ins2*^+/−^ mice had an increased lifespan in comparison to their *Ins2*^+/+^ littermates [81]. A strong association between insulin sensitivity and longevity was shown in Ames dwarf (missense mutation in Prop1 gene), growth hormone receptor knockout, or wild-type mice subjected to 30% CR [82]. Additional studies indicated that a reduction in insulin signaling could also have beneficial effects on lifespan [83,84,85,86]. Since CR can increase insulin responsiveness, its pro-longevity effects might be attributed to the changes in the insulin signaling pathway.

However, several studies on genetically modified mouse models demonstrated that increased insulin sensitivity is not required for CR to exert its beneficial effects on lifespan extension. For example, in mice lacking mammalian target of rapamycin complex 2 (mTORC2) activity in adipose tissue, CR still improved fitness and extended the lifespan of both males and females despite impaired insulin sensitivity [87]. Although most long-living rodent models have increased insulin sensitivity, some have normal or even decreased sensitivity [88], supporting the hypothesis that it is not required for lifespan extension. On the other hand, a partial disruption of the insulin receptor in mice’s peripheral tissues (liver, white adipose tissue, and skeletal muscle (PerIRKO^+/−^ mice)) did not affect lifespan, despite male mice showing enhanced hepatic insulin sensitivity [89].

Considering that CR induces a shift from carbohydrate to fat metabolism it can be assumed that the positive effects of CR on lifespan may be partially induced by an increase in fatty acid oxidation and ketogenesis [90]. Although not directly associated with the topic of this review, it is worth mentioning that the isocaloric ketogenic diet mimics the metabolic changes accompanying CR and significantly increases median lifespan and survival in mice [91].

### 2.2. CR, Endocrine Factors, and Longevity

The endocrine system integrates and coordinates adaptive response to CR, thus playing an important role in mediating CR-related pro-longevity effects. CR affects not only circulating insulin levels but also several other hormones and growth factors including growth hormone (GH), insulin-like growth factor 1 (IGF1), thyroid hormones, glucocorticoids, and reproductive hormones, all of which possibly influence longevity. Various CR interventions were shown to decrease GH, IGF1 [92], thyroid [93,94,95], and reproductive hormone circulating levels [96]. By contrast, glucocorticoids were increased by CR. CR restores plasma concentrations of some hormones to levels characteristic for younger individuals whereas, paradoxically, other changes resemble aging [96].

Metabolic changes induced by CR are mainly mediated by hormones that induce cellular alterations in energy consumption, ultimately diminishing overall energy requirements [97]. The reduction in thyroid hormones observed following CR lowers metabolic rate and core body temperature [97], enabling energy preservation. A decrease in triiodothyronine (T3), usually accompanied by unchanged levels of serum thyroxine (T4) and thyroid-stimulating hormone (TSH), was reported in several human CR studies [41,51,93,94,95,98]. Similarly, the CRONIES (a group of people self-imposed on long-term CR) have significantly lower levels of serum T3, but not T4 and TSH, compared to controls [99]. Lower plasma levels of thyroid hormones and higher plasma TSH levels were observed in long-lived mutant mice and centenarians [100], suggesting a potential role of lower thyroid activity in longevity. Furthermore, a population-based study carried out in the Netherlands showed that individuals with low–normal thyroid function live up to 3.5 years longer than those with normal–high thyroid function [101]. Therefore, a CR-mediated decrease in thyroid hormones might be involved in lifespan extension by reducing metabolism rate, oxidative stress, and cell senescence [102].

Downregulation of GH/IGF1 production is associated with increased longevity in many animal models. Moreover, CR-induced reduction in serum IGF1 levels in animals is related to a longer lifespan [92,103]. However, in humans, aging is associated with a decrease in GH and IGF1, resulting from the decreased amount of GH secreted without changing the frequency of GH secretion [99]. Circulating GH concentration in humans is unaltered after six months of CR [99]. The same applies to plasma IGF1 level after one or two years of moderate CR [41]. In any case, the plasma concentration of IGF1 in centenarians is similar to that in aged subjects [104]. Diverse results in animal and human studies suggest that the relationship between IGF1 status and longevity is complex and does not follow a simple correlation principle.

Plasma DHEAS concentration, the aforementioned endocrine marker of longevity, is also known to decline during aging. It was shown that CR could attenuate its age-associated decline [99]. Although the age-associated decline in plasma DHEAS could be slowed down by long-term CR in young adult rhesus monkeys [105], CR was shown to be ineffective on this ground in older rhesus monkeys [106] and a similar pattern was observed in humans [48]. Additionally, it was shown that the plasma concentration of DHEAS in centenarians is not different from that measured in aged subjects [107]. Altogether these studies question the role of plasma DHEAS in CR-mediated pro-longevity effects.

### 2.3. CR, Immune Function, and Longevity

Chronic, low-grade inflammation observed in the elderly can lead to various age-related diseases [108]. Senescent cells accumulating with age release multiple proinflammatory molecules such as tumor necrosis factor (TNF), interleukin 1β (IL-1β), IL-6, monocyte chemoattractant protein 1 (MCP-1), macrophage inflammatory protein 1α (MIP-1α), RANTES, and IL-18 [108]. Considering that CR delays and/or prevents most of the typical age-related chronic diseases, numerous studies looked into the link between CR and enhanced immune function [108].

CR exerts anti-inflammatory effects both by attenuating the age-related increase in pro-inflammatory mediators and by regulating the activity of their upstream signaling pathways. Some of the pro-inflammatory molecules regulated by CR are nuclear factor-κB (NF-κB), IL-1β, IL-6, TNF, cyclooxygenase 2 (COX-2), and inducible nitric oxide synthase (iNOS) [108,109,110]. Namely, even 10-day moderate CR is effective in changing the expression of pro-inflammatory transcription factors, NF-κB and activator protein 1 (AP-1), and associated genes in the kidneys of 24-month-old rats [109]. Chronic CR can ameliorate the age-related decrease in peroxisome proliferator-activated receptor α (PPARα) and PPARγ mRNA and protein content, factors that play important role in the immune response by inhibiting the expression of inflammatory cytokines [111]. Sixteen weeks of mild CR (15% reduction) attenuated the expression of inflammatory cytokines in adipose tissue [77]. Moreover, plasma C-reactive protein (CRP) and TNF-α levels decreased after long-term moderate CR in non-obese humans [110] while serum lipopolysaccharide-binding protein (LBP) and TNF-α were lower following two weeks of CR in mice [112]. The aforementioned findings imply that the duration and intensity of CR play a crucial role in its overall effect on the immune system in particular and lifespan in general.

Furthermore, CR affects the expression of various regulators of energy metabolism and stress response, which can indirectly affect the activity of some pro-inflammatory agents [108]. Emerging data indicate that adipose tissue might play a key role in the integration of metabolism and inflammation [113]. For example, CR upregulates serum adiponectin in humans [78] and mice [77], and it was reported that this adipocyte-derived hormone exerts an anti-inflammatory effect by suppressing the synthesis of TNF and interferon-gamma (INFγ) [77]. Therefore, a CR-mediated increase in adiponectin may account for the anti-inflammatory effects of CR. Moreover, myriad other genes involved in the regulation of energy metabolism are upregulated by CR, and the expression of more than 50 pro-inflammatory genes is reduced [114]. CR’s ability to oppose a wide range of age-associated diseases, such as cancers and cardiovascular disease, might be underlain by the reduction in systemic inflammatory tone [113].

Interestingly, CR exerts distinct effects on adipocyte-derived cytokines in obese and lean mice. Although a 30% reduction in food intake decreased the expression of IL-6, IL-2, IL-1Rα, MCP-1, and chemokine ligand 16 (CXCL16) in the adipose tissue of obese mice, the opposite effects were observed in lean mice [115]. The importance of this finding is reflected in the fact that elevation in plasma of levels of several inflammatory cytokines (such as IL-6, IL-18, IL-15, CRP) in centenarians was counterbalanced by a concomitantly increased concentration of anti-inflammatory molecules [75].

Lastly, anti-inflammatory effects related to CR are not restricted to the adipose tissue. A CR-induced increase in plasma corticosterone level is important in mediating CR anti-inflammatory effects by suppressing the systemic inflammatory response [116].

### 2.4. CR, Gut Microbiota, and Longevity

The effect of aging on gut microbiota composition was studied in fish, mice, rats, and humans. During aging, microbial communities become less diverse, and the number of pathogenic bacterial species increases [117,118,119,120]. In the gut microbiota of young (20-week-old) mice, *Akkermansia* and *Parabacteroides* are abundant, while in aged (100-week-old) mice their relative abundance decreases concurrently with an increase in *Turicibacter* and *Helicobacter* [120]. Moreover, some gut microbial metabolites are significantly different between young and aged mice. For example, reductions in short-chain fatty acids (SCFAs) (such as butyrate) and γ-aminobutyric acid (GABA) biosynthesis occur during aging [120]. Interestingly, centenarians have a specific composition of gut microbiota characterized by increased diversity and richness in health-associated and young-specific bacteria [75]. For example, the microbiota of Italian semi-supercentenarians is enriched in health-associated bacterial genera including *Akkermansia*, *Bifidobacterium*, and *Christensenella* [121]. Similarly, the microbiota of Spanish centenarians contains an abundance of the genera *Klebsiella, Lactobacillus, Parabacteroides*, and *Akkermansia* [122]. *Bacteroides fragilis*, *Parabacteroides merdae*, *Ruminococcus gnavus* and *Clostridium perfringens* are taxa identified in the microbiota of Chinese centenarians that may contribute to their longevity [123].

Studies using fecal microbiota transfer (FMT) highlighted the importance of microbiota in extending lifespan in various animal species. Microbiota transplantation from young into old fish significantly increases lifespan [118]. In addition, transplantation of microbiota from long-living people to mice increases the number of beneficial bacteria and lowers metabolites related to aging [124]. Furthermore, fecal transplantation of healthy microbiota can significantly improve the health and extend the lifespan of progeroid mice [122], suggesting a protective role of microbiota against accelerated aging. Moreover, *Akkermansia muciniphila* transplantation was sufficient to exert beneficial effects on lifespan extension in the mouse model of progeria (13.5% increase in median lifespan and 9% in maximal survival) [122]. Oral administration of *A. muciniphila* diminished the age-related deterioration in intestinal barrier function and reduced proinflammatory cytokines in C57BL/6J mice [125], and increased intestinal concentrations of several anti-aging metabolites, including bile acids, SCFAs, 2-hydroxybutyrate and polyamines [123].

The exact mechanism of microbial impact on host longevity is still poorly understood. However, it was shown that the genetic composition of gut microbes and increased production of some bacterial metabolites, such as polysaccharide colanic acid, affect host longevity in *C. elegans* as well [126]. Metabolome profiling analysis of ileal content from progeroid mice after FMT of wild-type microbiota point to an increase in secondary bile acids and other metabolites such as arabinose, ribose, and inosine as potential mediators accounting for health span and lifespan improvement [122].

It was shown that CR can induce taxonomic and functional changes in the microbiome. Wang and colleagues used FMT intervention to demonstrate that gut microbiota plays an important role in CR-mediated metabolic improvement [127]. Other studies also showed that CR can significantly alter mouse and human gut microbiota [127,128,129,130] by increasing the relative abundance of probiotic and butyrate-producing microbes and decreasing the abundance of proinflammatory species [128]. Different CR interventions in rats and mice enriched microbial phylotypes that positively correlated with longevity, which belonged mostly to Firmicutes, such as *Lactobacillus* [129,130,131]. Phylotypes positively correlated with lifespan contain beneficial bacteria that can protect the host from gut barrier disruption induced by different pathogens and reduce inflammatory cytokines. On the other hand, CR limits phylotypes Bacteroidetes, Firmicutes, Proteobacteria, and Actinobacteria, which are negatively associated with lifespan [129]. For example, CR mice have the lowest level of phylotypes in the Streptococcaceae family, of which some species can induce mild inflammation [129]. Although *Lactobacillus* is not predominant in aged mice, its abundance increases after CR [128]. Only two weeks of CR is sufficient to increase *Lactobacillus* abundance in mouse microbiota [112]. Additionally, a 30% CR diet significantly increased *Lactobacillus* and *Bifidobacterium* while simultaneously decreasing *Helicobacter* [127]. Moreover, CR promotes a reduction in the Firmicutes/Bacteroidetes (F/B) ratio, which increases from birth to adulthood, where a high F/B ratio is associated with dysbiosis [129,131]. Some beneficial effects of CR may be explained by an increased relative abundance of probiotic strains that protect against pathogen-induced gut barrier disruption, inhibit pathogen adhesion to the intestinal wall, and reduce inflammatory cytokines [128]. It was proposed that *Lactobacillus murinus* protects the gut barrier and attenuates chronic systemic inflammation in CR mice [112]. In addition to the induced changes in intestinal microbiota communities, CR was associated with the modulation of microbiota-derived metabolites [128] implicated in the regulation of intestinal function and barrier integrity [119,132]. The beneficial effects of the intestinal microbiota are mainly mediated via SCFAs [132], whose production is known to decline during aging [133]. On the other hand, a relative increase in abundance of butyrate-producing bacteria in centenarians is reported [132]. The improved gut barrier was observed in obese women after four weeks of a very-low-calorie diet [78]. Some of the described changes in bacterial communities and metabolites detected after CR interventions in murine models are comparable with those seen in centenarians. Although there is high variation in microbial communities among different centenarian populations, a unique shift in intestinal microbiota, characterized by enrichment in health-associated bacteria (such as *Lactobacillus* and *Akkermansia*) and reduction in taxa related to chronic inflammation seems to be associated with longevity [132]. Similar microbial taxonomic features and increased diversity found in CR animals and centenarians suggest that alteration in intestinal microbiota induced by CR might underlie the pro-longevity effects of this intervention. This notion is further emphasized by the fact that CR increased lifespan in Lobund–Wistar rats, but had no such effect on germ-free animals [134].

Taken together, available data indicate that alterations induced in the intestinal microbiota by CR might contribute to extending lifespan and health span, thus delaying the onset of age-associated diseases.

## 3. Molecular Mechanisms Underlying CR Effects on Health and Lifespan

Deciphering a molecular basis of CR effects is under continuous investigation to provide new directions for developing strategies that promote healthy aging. Thus far, several nutrient-sensing pathways and their key components involved in the pro-longevity effects of CR were described (reviewed in [135,136]). These nutrient-sensing mechanisms include insulin/IGF-1, AMP-kinase (AMPK), mTOR kinase, and sirtuin signaling.

### 3.1. Insulin/IGF1 Signaling

Insulin/IGF1 signaling is initiated by activation of their cognate receptors, which are highly homologous and share numerous common downstream effectors. Intracellular signal transduction from these receptors includes the stimulation of various signaling pathways involved in the regulation of metabolism, cell growth, and differentiation [137,138].

The hypothesis that downregulation of insulin/IGF1 signaling can extend lifespan is supported by various studies in genetically modified model organisms [139,140,141,142] and multiple epidemiological studies in humans [143,144,145,146]. Additionally, genetic modulations described as beneficial for the extension of mammalian lifespan include the deletion of several insulin/IGF1 signaling effectors, such as *Irs1* [84], S6 kinase 1 (S6K1) [147], Akt1 [86]. Meanwhile, FOXO proteins, activated upon reduced insulin/IGF1 signaling, were described as mediating CR pro-longevity effects [148]. It should be noted that, in mammals, a global reduction in a specific component of the insulin/IGF1 signaling pathway appears to be less effective in promoting longevity compared to invertebrate model organisms. Moreover, specific manipulation of this pathway in individual tissues, such as neural [85,149] and adipose tissue [83], was described as more beneficial.

### 3.2. AMPK

One of the downstream effectors of the insulin/IGF1 pathway and also a pro-longevity mediator is AMPK, which is activated under nutritional stress to maintain cellular and thus energy homeostasis as well [150]. This energy-sensing kinase consists of diverse components, with multiple isoforms that are organized in heterotrimeric complexes with one catalytic and two regulatory subunits [151,152,153], making its function versatile and its regulation complex.

In general, AMPK acts as a major regulator of cellular metabolic flexibility by simultaneously promoting and inhibiting processes involved in ATP synthesis and hydrolysis, respectively [154]. AMPK is activated upon an increase in the AMP/ATP ratio; activated AMPK turns on catabolic pathways to restore ATP levels by promoting glycolysis and fatty acid oxidation and by using mitochondrial substrates as energy sources (reviewed in [155]).

Furthermore, AMPK also controls cellular lipid metabolism by activating fatty acid uptake and oxidation while simultaneously inhibiting their synthesis [154].

Genetic AMPK activation in invertebrate organisms [156,157,158] and its pharmacological activation via metformin treatment in mice [155,159,160] were implicated in prolonging lifespan. However, systemic AMPK activation in rodents was shown to display somewhat adverse effects [161], which is not surprising considering its structural and functional heterogeneity. Distinct regulation of this metabolic regulator was documented in numerous studies reporting contrasting outcomes of various models of CR on tissue-specific AMPK activity [162,163,164,165]. Furthermore, recent studies reported that more subtle activation of AMPK, restricted to selected tissues, appears to be sufficient for transducing the kinase’s pro-longevity effects [166,167].

Several mechanisms underlying CR-mediated AMPK activation were reported, including regulation of SIRT-1 activity, members of the FOXO family, and mTOR signaling (reviewed in [155]).

### 3.3. mTOR

Another major metabolic regulator that is modulated by feeding regimes is mTOR protein kinases. These enzymes include two structurally and functionally distinct complexes, mTORC1 and mTORC2, which respond to a plethora of environmental inputs including nutrient availability, and are involved in the regulation of cell growth and survival, as well as protein and lipid synthesis [168]. Genetic or pharmacological inhibition of the mTOR pathway was shown to prolong life expectancy in worms [169,170], flies [171,172], and mice [173,174]. On the other hand, limited nutrient availability was reported to result in mTOR inhibition via several upstream signaling pathways involving PI3K, AMPK, and RAGs [175]. The mTOR pathway was implicated in the regulation of several hallmarks of aging, such as autophagy, protein synthesis, and mitochondrial and stem cell function [176].

### 3.4. Sirtuins

Sirtuins are histone deacetylases whose activity is dependent on nicotinamide adenine dinucleotide (NAD+), and thus tightly linked to cellular energy status [177]. In mammals, the SIRT family includes seven members (SIRT1–7) implicated in diverse cellular processes including metabolism, oxidative stress, cell survival, and autophagy in various tissues. Many members of this family were reported to exert additional enzymatic activities, exhibit diverse subcellular localizations and target numerous proteins, making them involved in multiple regulatory mechanisms [178].

Various reports suggested that aging is associated with a decline in NAD+ levels [179]. Meanwhile, NAD+ levels and sirtuin activity were observed to increase with fasting, supporting the hypothesis that sirtuins and NAD+ mediate the positive effects of CR on metabolism and longevity [180]. Interestingly, all enzymes involved in the control of cellular metabolic processes are acetylated [181], implying that sirtuins exert a major role in the modulation of these processes.

Consequently, it was demonstrated that under nutrient-deprived conditions, SIRT1 represses PPARγ transactivation activity, thus leading to the downregulation of adipogenesis [182]. In skeletal muscle, nuclear SIRT1 activates PGC-1a, consequently increasing mitochondrial fatty acid oxidation [183]. The reduced oxidative stress reported in mice fed with a restricted caloric diet was associated with increased SOD2 activity mediated by mitochondrial SIRT3 [184]. In pancreatic beta cells, SIRT1 promotes insulin secretion by repressing UCP2 [185]. In hepatocytes, sirtuin activation promotes the FOXO1-dependent transcription of genes related to gluconeogenesis and glucose release [186]. Additionally, SIRT1 deacetylates a key lipogenic activator, SREPB1c, thus reducing its affinity for promoters of the target genes involved in fat synthesis in the liver [187].

One of the hallmarks of aging is impaired autophagic function, resulting in the accumulation of dysfunctional cellular proteins [188]. Various members of the sirtuin family were shown to modulate proteins involved in autophagic pathways [189].

### 3.5. Cross-Regulation of Molecular Mechanisms

**Figure 1:**
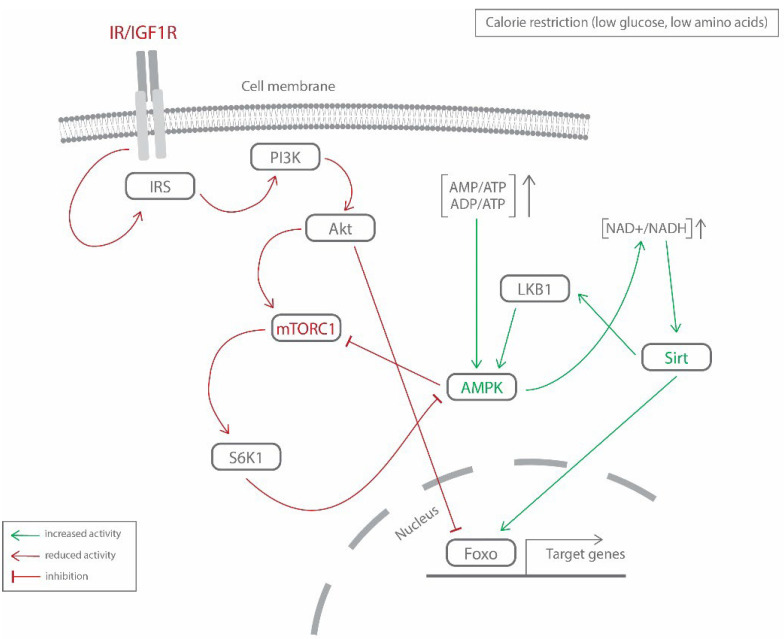
The interplay of the major nutrient-sensing pathways (IR/IGF1, mTORC1, AMPK, sirtuins) underlying CR’s pro-longevity effects. This figure represents a simplified version of the interconnectivity between the main molecular mechanisms and their components addressed in this review. IR/IGF1R—insulin/insulin-like growth factor 1 receptor; IRS—insulin receptor substrate; PI3K—phosphoinositide 3-kinase; Akt—protein kinase B; mTORC1—mammalian target of rapamycin complex 1; S6K1—S6 kinase 1; AMPK—AMP-activated protein kinase; LKB1—liver kinase B1; Sirt—sirtuins; Foxo—forkhead box protein O.

It is apparent from previous sections that the molecular mechanisms underlying CR’s beneficial effects on lifespan are complex. Furthermore, multiple studies revealed the interconnectivity of various nutrient-sensing pathways, highlighting the intricate regulation of favorable pro-longevity outcomes (Figure 1).

In nematodes, AMPK acts as an effector of DAF-16/FOXO-mediated effects on longevity [157,190]. Deletion of ribosomal protein S6K1, a common downstream effector of TOR and insulin/IGF1 signaling, was demonstrated to result in lifespan extension in mice. Additionally, observed gene expression changes in mice lacking SK61 were similar to those detected under CR treatment, including increases in AMPK expression and activity [147]. Furthermore, AMPK acts as an upstream suppressor of mTOR signaling [175]. Employing the systems biology approach, Hou and coworkers uncovered that, in nematodes, simultaneous modulation of three age-associated genetic modules (mTOR, AMPK, insulin-IGF1 signaling/FOXO) is required to recapitulate CR’s beneficial effects on longevity [191]. Multiple studies indicated that AMPK and sirtuins require one another to regulate metabolic homeostasis by promoting each other’s activity [192].

### 3.6. New Determinants (Multi-Omics Approach)

An additional level of intricacy in CR-mediated pro-longevity effects results from the inter-tissue communication mediated by molecules secreted into systemic circulation. The development of high-throughput technologies allowed researchers to discover novel CR mediators by the simultaneous investigation of millions of biological molecules. Recently, Ma et al. presented a multiple tissue single-cell transcriptomic atlas to study aging and its modulation by caloric restriction [193]. They observed a reduction in age-induced systemic inflammatory response upon CR due to the decreased number of plasmocytes in various tissues and induced modulation of macrophages towards an anti-inflammatory state. Aging was associated with a decline in multiple miRNA levels [194,195,196], while CR was shown to modulate miRNAs correlated with insulin sensitivity, adiposity [197], neuroprotection [198,199], and mitochondrial homeostasis [200]. Furthermore, caloric restriction was reported to affect the expression of various genes via DNA methylation in numerous model systems [201,202,203,204]. Most recently, a combined study of physiological and multi-omics data (transcriptomics, metabolomics) identified the potential involvement of the glycine–serine–threonine metabolic axis and the metabolism of short-chain and polyunsaturated fatty acids in delaying aging, thus promoting longevity [205].

## 4. Discussion

Whether or not CR prolongs human life is a question that still remains without a straightforward answer. Many issues still need to be clarified. For example, there are uncertainties regarding whether a decrease in calorie intake alone or a specific dietary pattern that takes into account both energy content and dietary macronutrient composition is more effective in terms of prolonging health span (reviewed in [206]). The latter approach is also supported by the findings from centenarian studies, where dietary characteristics other than the reduced amount of food/calories were shown to contribute to their exceptional longevity. For example, Okinawa’s dietary pattern was shown to partially overlap with other healthy nutritional styles, such as the Mediterranean diet (MedDiet) known for its low saturated fat content, high antioxidant intake, low glycemic load, and components that can be characterized as “functional foods”, all of which reduce the risk of main age-related diseases [207]. The Mediterranean diet influences factors that determine life- and health span, including plasma lipoprotein levels, insulin resistance, hormonal status, antioxidant defense capacity, cardiovascular health and many others (reviewed in [208]). A meta-analysis of the MedDiet and its components in relation to all-cause mortality indeed indicated a beneficial association of the MedDiet with health status [209] and longevity. It also pointed to specific dietary components that mostly influence this association [210]. Namely, consumption of fruits/nuts and vegetables showed the highest inverse association with all-cause mortality. A subsequent study confirmed that the risk of all-cause mortality linearly decreased with the increase in adherence to a MedDiet [211].

Recent systematic review and meta-analysis of controlled trials in adults confirmed the benefits of the MedDiet on metabolic health and suggested the need to promote this dietary pattern to adult populations [212]. Other studies also indicated the MedDiet approach to be appropriate for the elderly to adopt/preserve in order to maximize health and prolong lifespan [213]. Moreover, efforts were made to implement a Mediterranean-style diet outside the Mediterranean region [214].

All studies mentioned above impose the question of what the best dietary approach would be for prolonging lifespan, and if the same approach can be applied for healthy, non-obese individuals and for those who are overweight. Some CALERIE data question the sustainability of some of the CR-induced beneficial effects and indicate that to preserve them after weight stabilization, sharp CR would most likely be constantly required [59]. Long-term adherence to sharp CR is not something many of us would happily agree to, even if we knew that such a regimen would prolong our lifespan.

Additionally, the beneficial effects of CR in Okinawan subjects were further challenged by the possibility that the longevity advantage observed in this population was lost for the generations born after World War II. Le Bourg suggests that the Okinawan population is an example of how severe malnutrition can be harmful to later generations as well [215]. Namely, Le Bourg stresses that (at that time) there were no data on humans with a normal body mass index (neither overweight nor obese) that clearly demonstrated the life extension effect of CR. He supports the idea that studies testing the effects of CR in humans should focus on the health effects in overweight and obese people, rather than normal-weight people [215] and advocates that CR would probably not increase human lifespan in normal-weight subjects [216]. It would also be interesting to see if/how a change in traditional diets (such as the Mediterranean) and the inevitable evolution of dietary patterns observed in some Blue Zones would influence lifespan. The Mediterranean food tradition considered responsible for many beneficial effects and perhaps even for longevity in Italy and Greece has undergone a significant change [33]. Unfortunately, we are decades away from obtaining such data and making a definitive conclusion.

## 5. Conclusions

Current literature unfortunately cannot offer a definitive answer to the question asked in the title of this manuscript. The existing data regarding nutritional habits/caloric intake of the centenarians in LBZs are not reliable and/or detailed enough to provide a direct causal link between caloric intake and longevity in humans, while the results on lifespan from other studies in humans may never be available. Non-human primates’ studies gave contradictory results. However, nutrition undoubtedly has profound effects on aging, and more certainly and importantly, on health span. A comprehensive study investigating adherence to the World Health Organization (WHO) guidelines for a healthy diet to prevent chronic diseases and postpone death showed that such dietary regimens increased life expectancy by two years at the age of 60 years and was associated with greater longevity in elderly men and women in Europe and the United States [217].

Dozens of studies mentioned herein showed that longevity in humans is associated with a significant improvement in glucose handling and insulin sensitivity and with a decline in plasma IGF1 levels. That CR is currently the best-known positive modulator of these metabolic processes speaks again in favor of its potential to fight obesity and metabolic diseases and to promote healthier feeding habits in modern society, and thus to increase longevity. However, greater caution is needed, as CR could have adverse effects and thus as an approach is inadvisable to start in aged humans. Higher frailty levels and increased anxiety were detected in experimental models of late-onset CR [68,69,70] and similarly could happen in older people who are often at risk of undernutrition and sarcopenia, rather than of obesity, and in those who already have an appropriate diet and a normal weight. Instead of a simple decrease in calorie intake, a specific longevity diet that encompasses a broader range of nutritional aspects was proposed as a multi-pillar approach adjusted for the age and health status of the subject (reviewed in [2]).

In this manner, a reduction in calories as one component of a healthy nutritional approach could represent a valuable component of standard healthcare and could be a preventative antiaging measure.

## Figures and Tables

**Table 1:** Most relevant studies on human longevity.

Study	Population	Topic
[16]	NA	Centenarian offspring as a model for understanding longevity
[20]	Okinawan population	Genetic aspects of longevity
[21]	Sardinian population	Clustering in Sardinian longevity
[22]	Okinawan population	Nutritional status in Okinawan centenarians
[23]	Sardinian population	Food habits and lifestyle in Sardinian centenarians
[24]	School children in Okinawa	Nutrition factors and longevity
[25]	Japanese population	Impact of westernization on the nutrition of Japanese
[26]	Ikarian population	Centenarians’ health status
[27]	Ikarian population	Lifestyle and longevity
[28]	Italian centenarians	Gender differences in longevity
[29]	Costa Rican/Sardinian centenarians	Dietary habits and longevity
[30]	Japanese centenarians	Factors associated with longevity in Japan/ 1990 population census data
[31]	Ikarian population	Sociodemographic and lifestyle statistics of Ikarian old people (>80 years)
[32]	Sardinian male population	Y Chromosome markers and centenarians/genetic structure of the Sardinian population
[33]	Sardinian population	Evolution of the Sardinian dietary patterns
[34]	Sardinian population/AKEA study	Identification of Sardinia island as an area characterized by extreme longevity
[18]	Blue Zones	Blue Zones as areas of exceptional longevity
[19]	Greek Adults/EPIC Study	Hypertension in the Greek general population
[17]	NA	Worldwide demography of centenarians
[7]	New England population	Aging rate of centenarians
[35]	Okinawan population	Caloric restriction and human longevity
[36]	Okinawan population	Caloric restriction/Okinawan diet
[37]	Okinawan and American septuagenarians	Caloric restriction in Okinawans and Americans
[38]	Okinawan population	Validation study of centenarians in Okinawa
[39]	Okinawan and Japanese populations	Demographic, phenotypic, and genetic characteristics of centenarians in Japan

**Table 2:** The changes in various physiological factors detected in animals and humans during ag-ing and under CR. ↓ decrease; ↑ increase; − without change; IGF1—insulin-like growth factor 1; GH—growth hormone; T3—triiodothyronine; T4—thyroxine; TSH—thyroid-stimulating hormone; DHEAS—dehydroepiandrosterone sulfate.

Physiological Factor	Detected during Aging in Animals and Humans	Detected during CR in Animals and Humans	Detected in Centenarians
Insulin sensitivity	↓	↑	↑
Circulation insulin	↑	↓	↓
Fasting blood glucose	↑	↓	↓
Serum IGF1	↓	↓ −	−
Serum GH	↓	−	
Serum glucocorticoids	↑	↑	
T3		↓	↓
T4	−	−	↓
TSH	↑	−	↑
DHEAS	↓	↑ −	−
Inflammation	↑	↓	↑↓
Gut microbiota diversity	↓	↑	↑

## Abbreviations

ADP	adenosine diphosphate
Akt1	protein kinase B
AMP	adenosine monophosphate
AMPK	AMP-activated protein kinase
ATP	adenosine triphosphate
CALERIE	Comprehensive Assessment of Long-term Effects of Reducing Intake of Energy
COX-2	cyclooxygenase 2
CR	caloric restriction
CRP	C-reactive protein
CXCL16	chemokine ligand 16
DHEA	dehydroepiandrosterone
DHEAS	dehydroepiandrosterone sulfate
FMT	fecal microbiota transfer
FOXO	forkhead box protein O
GABA	γ-aminobutyric acid
GH	growth hormone
GLUT	glucose transporter
HDL	high density lipoprotein
IGF1	insulin-like growth factor 1
IL	interleukin
INFγ	interferon gamma
iNOS	inducible nitric oxide synthase
Irs, IRS	insulin receptor substrate
LBP	lipopolysaccharide—binding protein
LBZ	Longevity Blue Zones
LDL	low dencity lipoprotein
LKB1	liver kinase B1
MCP-1	monocyte chemoattractant protein 1
MedDiet	Mediterranean diet
MIP-1α	macrophage inflammatory protein 1α
mTOR	mammalian target of rapamycin kinase
mTORC	mTOR complex
NAD+	nicotinamide adenine dinucleotide
NF-κB	nuclear factor-κB
NIA	National Institute on Aging
PGC-1a	peroxisome proliferator-activated receptor gamma coactivator 1-alpha
PI3K	phosphoinositide 3-kinase
PPAR	peroxisome proliferator-activated receptor
RAGs	recombination-activating genes
RANTES	regulated on activation, normal T cell expressed and secreted
S6K1	S6 kinase 1
Sirt, SIRT	sirtuins
SOD2	superoxide dismutase 2
SREPB1c	sterol regulatory element-binding protein 1c
T3	triiodothyronine
T4	thyroxine
TNF	tumor necrosis factor
TNO	Toxicology and Nutrition Institute in the Netherlands
TSC2	tuberous sclerosis complex 2
TSH	thyroid stimulating hormone
UCP2	mitochondrial uncoupling protein 2
WHO	World Health Organization
